# Echo Time Dependency of Local Activity Metrics of Resting-State Functional MRI

**DOI:** 10.3389/fnins.2021.619412

**Published:** 2021-03-16

**Authors:** Li-Xia Yuan, Na Zhao, Xiu-Qin Wang, Ya-Ting Lv, Hongjian He

**Affiliations:** ^1^Center for Cognition and Brain Disorders, The Affiliated Hospital of Hangzhou Normal University, Hangzhou, China; ^2^Institute of Psychological Sciences, Hangzhou Normal University, Hangzhou, China; ^3^Zhejiang Key Laboratory for Research in Assessment of Cognitive Impairments, Hangzhou, China; ^4^Unit of Psychiatry, Faculty of Health Sciences, Center for Cognition and Brain Sciences, Institute of Translational Medicine, University of Macau, Macao, China; ^5^Center for Brain Imaging Science and Technology, College of Biomedical Engineering and Instrumental Science, Zhejiang University, Hangzhou, China

**Keywords:** echo time, amplitude of low-frequency fluctuation, fractional amplitude of low-frequency fluctuation, regional homogeneity, degree centrality, resting-state fMRI

## Abstract

Local activity metrics of resting-state functional MRI (RS-fMRI), such as the amplitude of low-frequency fluctuation (ALFF), fractional ALFF (fALFF), regional homogeneity (ReHo), and degree centrality (DC), are widely used to detect brain abnormalities based on signal fluctuations. Although signal changes with echo time (TE) have been widely studied, the effect of TE on local activity metrics has not been investigated. RS-fMRI datasets from 12 healthy subjects with eyes open (EO) and eyes closed (EC) were obtained with a four-echo gradient-echo-planar imaging pulse sequence with the following parameters: repetition time/TE1/TE2/TE3/TE4 = 2,000/13/30.93/48.86/66.79 ms. Six representative regions were selected for simulating the spatial feature of TE dependency of local activity metrics. Moreover, whole-brain local activity metrics were calculated from each echo dataset and compared between EO and EC conditions. Dice overlap coefficient (DOC) was then employed to calculate the overlap between the *T* maps. We found that all the local activity metrics displayed different TE dependency characteristics, while their overall change patterns were similar: an initial large change followed by a slow variation. The *T* maps for local activity metrics also varied greatly with TE. For ALFF, fALFF, ReHo, and DC, the DOCs for voxels in four TE datasets were 6.87, 0.73, 5.08, and 0.93%, respectively. Collectively, these findings demonstrate that local metrics are greatly dependent on TE. Therefore, TE should be carefully considered for the optimization of data acquisition and multi-center data analysis in RS-fMRI.

## Introduction

Resting-state functional MRI (RS-fMRI) is a method used to record the blood oxygenation level-dependent (BOLD) signal changes caused by spontaneous neural activity (Biswal et al., 1995). Its widespread application in neuropsychiatry and cognitive neuroscience researches has made it the most predominant tool for investigating the features of brain baseline state (Damoiseaux et al., 2006). For instance, a segment of neuropsychiatric and neurocognitive studies often used local activity metrics of RS-fMRI to localize signal abnormalities (Zang et al., 2007; Zou et al., 2008; Anderson et al., 2014; Xu et al., 2015; Wang et al., 2017; Zhou et al., 2019). Commonly, local activity metrics are computed from the signal fluctuations. For example, the amplitude of low-frequency fluctuation (ALFF) and fractional ALFF (fALFF) characterize the low-frequency signal fluctuation (Yang et al., 2007; Zang et al., 2007; Zou et al., 2008), regional homogeneity (ReHo) describes the synchronization of signal fluctuations of several neighbored voxels (Zang et al., 2004), and degree centrality (DC) captures the synchronization of signal fluctuations from a certain voxel and the whole brain (Zuo et al., 2012).

Besides the brain’s spontaneous activity, RS-fMRI signal fluctuation is also dependent on physical measurement parameters, such as the echo time (TE). The TE dependence of signal intensity acquired with gradient-echo-planar imaging (GE-EPI) is usually approximated by a simple exponential decay. Usually, conventional EPI sequences measure the signal at a single TE, typically chosen to match the average apparent transverse tissue relaxation time ($T_{2}^{*}$) to maximize the BOLD contrast-to-noise ratio (CNR) (Bandettini et al., 1994; Krüger et al., 2001). Nonetheless, it is important to highlight that $T_{2}^{*}$ values vary to a great extent across the whole brain (Boulby and Rugg-Gunn, 2003; Yan et al., 2009), between subjects (Krüger and Glover, 2001), and also among field strengths (Bartzokis et al., 1993; Krüger et al., 2001; Boulby and Rugg-Gunn, 2003). As a result, the signal fluctuations observed in the brain inevitably reveal different BOLD contrasts. Consequently, the local activity metrics based on RS-fMRI signal fluctuations will also change with TE.

To date, only a few studies specifically investigated the TE dependence of the RS-fMRI metrics. One study, for example, found that the mean amplitude of normalized low-frequency fluctuations of intrinsic cerebral networks (e.g., motor and visual network) showed second-order polynomial dependence on TE (Wu et al., 2012). Moreover, the functional connectivity (FC) found broad and local correlations at short TE (TE ≤ 14 ms) and long-range connections at longer TE (TE ≥ 22 ms) (Wu et al., 2012). What is more, Peltier and Noll (2002) found that the FC of low-frequency fluctuations in RS-fMRI had a linear dependence on TE, whereas more recently, the BOLD response transients were demonstrated to be nonlinearly dependent on TE (Havlicek et al., 2017). However, TE’s influence on the local activity metrics (e.g., ALFF, fALFF, ReHo, and DC) has not been studied yet, even though it is essential for the optimization of data acquisition and multi-center data analysis in RS-fMRI, as a poor selection of TE can lead to false positive or false negative results and reduced reliability among studies.

To investigate the above-mentioned issue, the present study employed the multi-echo fMRI (ME-fMRI), a technique that simultaneously acquires multiple datasets with different TEs in a repetition time (TR). The time interval between the multi-TE datasets is usually less than 20 ms (Wu et al., 2012; Havlicek et al., 2017; Kundu et al., 2017), during which the physiological state and head motion can be assumed to be almost the same. Therefore, the ME-fMRI qualifies as the most appropriate method to study TE dependency of local activity metrics in RS-fMRI. Eyes open (EO) and eyes closed (EC) are two commonly used conditions in RS-fMRI, and the difference between EO and EC constitutes a within-group design, which is a widely used experiment design for exploring the variance within subjects in group-level analysis (Liu et al., 2013; Zou et al., 2015). Thus, in the current study, we employed ME-fMRI with four TEs and systematically investigated the TE influence on ALFF, fALFF, ReHo, and DC from two aspects: (1) simulating the TE dependency of local activity metrics with a small TE interval and a large TE range, then validating the simulation results with *in vivo* dataset, and (2) studying the TE influence on group-level statistical results of the local activity metrics with EO and EC ME-fMRI datasets.

## Materials and Methods

### Subjects and Data Acquisition

The experiment was approved by the Ethics Committee of the Center for Brain Imaging Science and Technology, Zhejiang University. Signed informed consent was obtained from all subjects before the data acquisition. Twelve healthy subjects (24.4 ± 1.8 years old, seven females) participated in the experiment. All subjects were pre-screened for any history of neurological illness or psychiatric disorders.

RS-fMRI datasets with EO and EC were obtained on a Siemens MAGNETOM Prisma 3T scanner (Siemens Healthcare, Erlangen, Germany). Before the scanning, all subjects were instructed to rest with their eyes open or closed, not to think of anything in particular, and not to fall asleep during scanning. The BOLD images were acquired with a four-echo gradient-echo-planar imaging (EPI) pulse sequence with the following parameters: TR/TE1/TE2/TE3/TE4 = 2,000/13/30.93/48.86/66.79 ms, flip angle = 78°, 20 slices with interleaved acquisition, slice thickness/gap = 4/1 mm, field of view = 220 × 220 mm^2^ with an in-plane resolution of 3.44 × 3.44 mm^2^, and 180 frames. The datasets with four different TEs were named as E1, E2, E3, and E4, respectively. The T_1_-weighted images were acquired using a 3D magnetization-prepared rapid-acquisition gradient echo sequence with a resolution of 1 × 1 × 1 mm^3^.

### Data Preprocessing

ME-fMRI data were preprocessed with SPM12 V7487^1^ and DPABI V2.1^2^. Preprocessing procedures included removal of the first 10 frames, slice-time correction, realignment for motion correction, and spatial normalization into the standard Montreal Neurological Institute (MNI) space with a voxel size of 3 × 3 × 3 mm^3^. The T_1_-weighted images were coregistered to the averaged functional image of E2 and segmented to obtain the forward deformation field. Then, head motion parameters and the forward deformation field from E2 were used for the other three datasets. The maximum translation and rotation were less than 1.5 mm and 1.5° for all the subjects. Lastly, data from two subjects were excluded from the analysis because their whole brain were not entirely scanned.

### Simulating the TE Dependency of Local Activity Metrics

#### $R_{2}^{*}\left(t\right)$ and *S_0_*(*t*) Calculation

After normalization, the signal intensities of each voxel from four different echoes at each frame were fitted to a mono-exponential signal decay with the least square method (Posse et al., 1999; Peltier and Noll, 2002):

(1) $$S\left({\text{TE}}_{n}\right)=S_{0}\text{exp}\left(-R_{2}^{*}\cdot {\text{TE}}_{n}\right),$$

where *S*_0_ is the initial signal, *n* is the number of TE, and *S*(TE_*n*_) is the signal at TE_*n*_. The natural log of the magnitude data with four echoes were fitted to a first-order polynomial. Then, $R_{2}^{*}\left(t\right)$ and *S*_0_(*t*) were calculated for all voxels.

#### Selection of Regions of Interest

To explore the spatial specificity of TE dependency of local activity metrics, i.e., ALFF, fALFF, ReHo, and DC, we selected six spherical seeds (6 mm in diameter) as the regions of interest (ROIs) from the default mode, dorsal attention, motor, visual, and auditory networks to conduct simulation (Van Dijk et al., 2010; Patriat et al., 2013). The seed coordinates were as follows: the posterior cingulate cortex (PCC) (0, −53, 26), the left hippocampal formation (HF) (−24, −22, −20), the left intraparietal sulcus (IPS) (−24, −58, 52), the left primary motor cortex (Mot) (−36, −25, 57), the left auditory cortex (Aud) (−43, 26, 12), and the left primary visual cortex (Vis) (−30, −88, 0).

#### Simulation of the TE Dependency of Local Activity Metrics

The curves of *S*_0_(*t*) and $R_{2}^{*}\left(t\right)$ of ROIs were extracted from a subject with EC. The $T_{2}^{*}$-weighted signal intensities of ROIs at different TEs were reconstructed with Eq. (1). TE was set to be 0:2:100 ms. The ALFF (0.01–0.08 Hz) were computed according to methodologies used in previous studies (Yang et al., 2007; Zang et al., 2007). The fALFF was acquired by normalizing ALFF with the averaged amplitude of the whole frequency band (Zou et al., 2008). The ReHo was computed as the Kendall coefficient of concordance (KCC) of the certain voxel and its 26 neighboring voxels at different TEs (Zang et al., 2004). The DC was calculated as the summed Pearson correlation coefficients (larger than 0.2) of the time series of a given voxel with that of each voxel in the whole brain (Zuo et al., 2012). Moreover, the local activity metrics from the four $T_{2}^{*}$-weighted images were calculated and compared with simulated results. Additionally, the simulation and comparison above were done for all the subjects under both EO and EC.

### TE Dependency of Local Activity Metrics of *in vivo* Dataset

#### ALFF and fALFF Calculation

Before ALFF calculation, the normalized RS-fMRI datasets were smoothed using a 3D Gaussian isotropic kernel [full width at half maximum (FWHM) = 6 mm]. Next, the signal was detrended and regressed out of head motion parameters (Friston24). For the standardization, both ALFF and fALFF of each voxel were divided by their global mean value to obtain mALFF and mfALFF.

#### ReHo Calculation

Before ReHo calculation, the normalized RS-fMRI datasets were detrended and regressed out of head motion parameters (Friston24) and then band-pass filtered (0.01–0.08 Hz). For the standardization, the ReHo of each voxel was divided by the global mean value to obtain the mReHo map, which was then smoothed (3D Gaussian isotropic kernel with FWHM = 6 mm) (Zang et al., 2004).

#### DC Calculation

Before DC calculation, the normalized RS-fMRI datasets were detrended, regressed out of head motion parameters (Friston24), and then band-pass filtered (0.01–0.08 Hz). For the standardization, the DC of each voxel was divided by the global mean value to obtain the mDC map, which was then smoothed (3D Gaussian isotropic kernel with FWHM = 6 mm) (Buckner et al., 2009; Zuo et al., 2012).

#### Statistical Analysis

Paired *T*-tests were performed between EO and EC on ALFF, fALFF, ReHo, and DC from E1–E4. The *T* maps were thresholded with *p* < 0.05 with cluster size > 50. The number of overlapped voxels across *T* maps from E1 to E4 was quantified with Dice overlap coefficient (DOC) (Dice, 1945). DOC was calculated as the voxel number of the intersection divided by the total voxel number in all thresholded *T* maps (Dice, 1945). Here, four thresholded *T* maps from E1 to E4 were overlapped together. The voxels could then be classified into any of the four following categories: (1) appeared in all the four *T* maps, (2) appeared in any three of the four *T* maps, (3) appeared in any two of the four *T* maps, and (4) appeared in only one of the four *T* maps. The total voxel number used for DOC is the summation of all four categories of voxels. The DOCs for voxels of (1)–(4) categories were abbreviated as DOC for 4 TEs, 3 TEs, 2 TEs, and 1 TE, respectively, and the summation of DOCs for 4 TEs, 3 TEs, 2 TEs, and 1 TE equals to 1.

## Results

### TE Dependency of Local Activity Metrics

**FIGURE 1:**
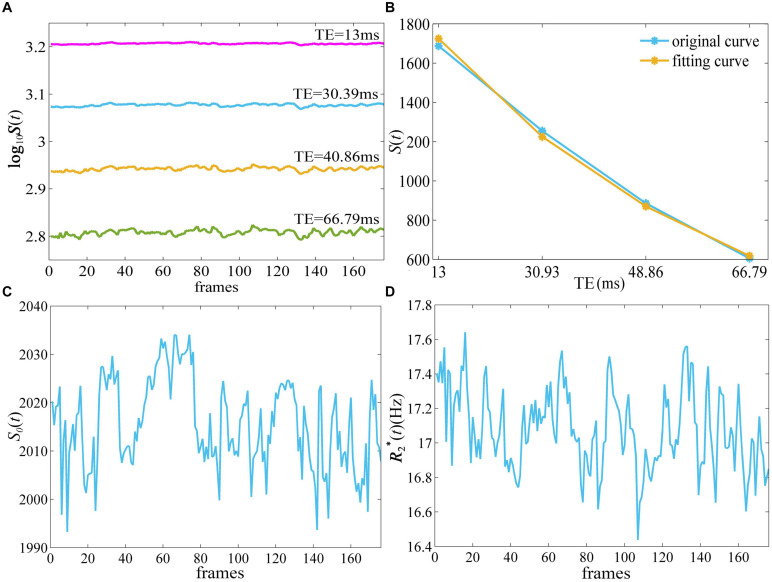
The derivation of quantitative $R_{2}^{*}\left(t\right)$ and *S*_0_(*t*) from ME-fMRI datasets. By fitting the signal intensities from four TEs **(A)** to a monoexponential model **(B)**, quantitative $R_{2}^{*}\left(t\right)$ **(D)** and *S*_0_(*t*) **(C)** were obtained. ME-fMRI, multi-echo fMRI; TEs, echo times.

Figure 1 depicts the derivation of *S*_0_ (*t*) and quantitative $R_{2}^{*}\left(t\right)$ from $T_{2}^{*}$-weighted images of ME-fMRI. The $T_{2}^{*}$-weighted signal intensity became smaller with larger TE, while the amplitude of fluctuation in the signal intensity at TE = 40.86 and 66.79 ms was more obvious than that at TE = 13 and 30.39 ms (Figure 1A). The $T_{2}^{*}$-weighted signal intensities from E1 to E4 of the first frame demonstrated an exponential decay (Figure 1B). When a mono-exponential model in Eq. (1) was applied to fit the signal, the *S*_0_ and $R_{2}^{*}$ of one frame can be obtained.

**FIGURE 2:**
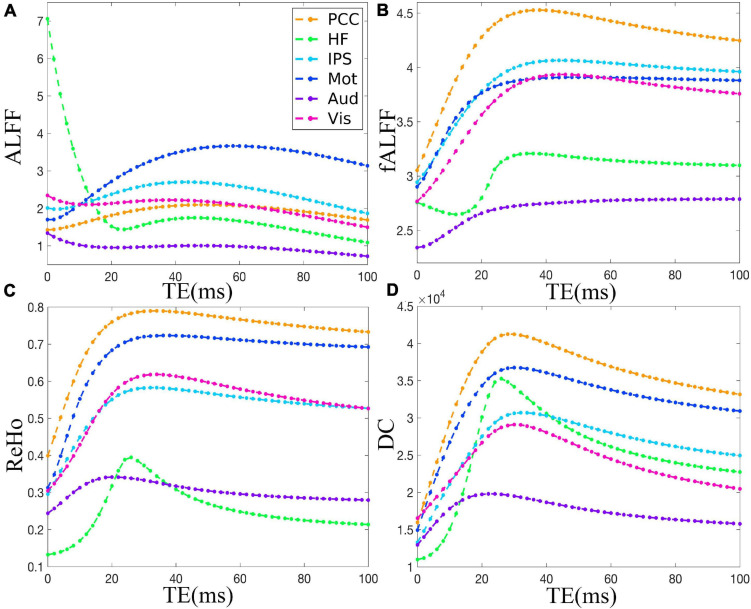
The simulation of the TE dependency of ALFF **(A)**, fALFF **(B)**, ReHo **(C)**, and DC **(D)** of six representative ROIs, i.e., PCC, HF, IPS, Mot, Aud, and Vis, from a dataset under eyes closed condition. ALFF, amplitude of low-frequency fluctuation; fALFF, fractional ALFF; ReHo, regional homogeneity; DC, degree centrality; ROIs, regions of interest; PCC, posterior cingulate cortex; HF, left hippocampal formation; IPS, left intraparietal sulcus; Mot, left primary motor cortex; Aud, left auditory cortex; Vis, left primary visual cortex.

**FIGURE 3:**
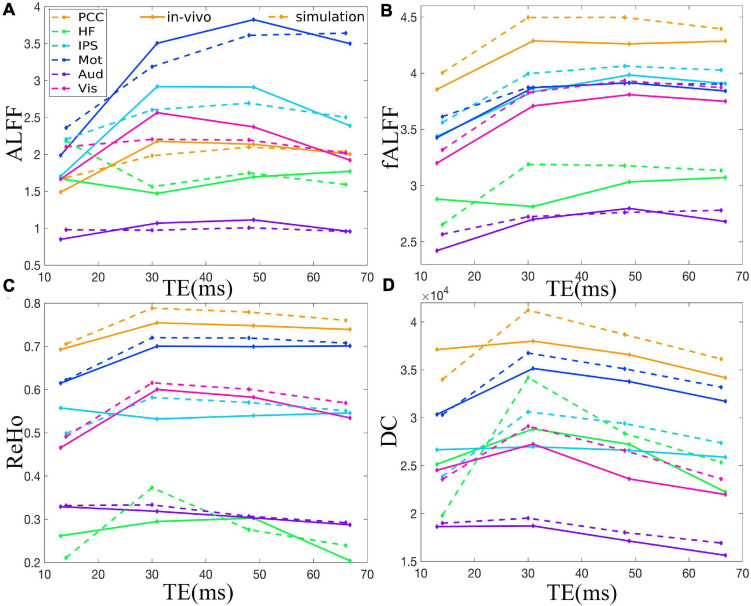
Comparison between simulation and *in vivo* value of ALFF **(A)**, fALFF **(B)**, ReHo **(C)**, and DC **(D)** of six representative ROIs from a dataset with EC, i.e., PCC, HF, IPS, Mot, Aud, and Vis. The solid and dotted lines denote the *in vivo* and simulation results, respectively. ALFF, amplitude of low-frequency fluctuation; fALFF, fractional ALFF; ReHo, regional homogeneity; DC, degree centrality; ROIs, regions of interest; PCC, posterior cingulate cortex; HF, left hippocampal formation; IPS, left intraparietal sulcus; Mot, left primary motor cortex; Aud, left auditory cortex; Vis, left primary visual cortex.

Figure 2 demonstrates that ALFF, fALFF, ReHo, and DC of all the ROIs displayed generally initial dramatic change. Nonetheless, soon, the four metrics entered a flat stage after reaching its maxima. Similarly, all the ROIs in the *in vivo* data were consistent with those of simulation (Figure 3). For ALFF, HF, IPS, and Vis initially displayed a descending trend but eventually rapidly ascending again. Once ALFF of HF, IPS, and Vis reached their maxima, a small decline was observed. As for both the Mot and PCC, we observed rising curves as TE became longer. In contrast, Aud kept decreasing across all the TEs (Figure 2A). For fALFF, PCC, IPS, Mot, Aud, and Vis, the four metrics rose dramatically until they reached their maxima. Finally, a small change was observed. Interestingly, only the HF experienced an initial decrease, followed by a pronounced rise and finally reaching a flat trend (Figure 2B). Similarly, the ReHo of all ROIs raised rapidly and reached their peaks when TE was 20∼30 ms, thereafter slowly decreasing (Figure 2C). Additionally, DC also revealed a consistent change pattern with the other three metrics. However, the variation of HF with TE was much larger when compared with that of ReHo (Figure 2D).

**FIGURE 4:**
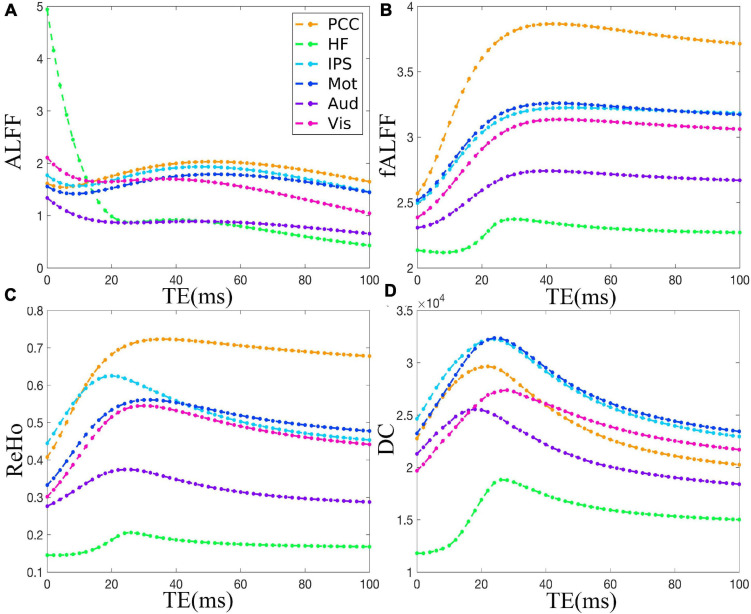
The simulation of the TE dependency of the group-averaged ALFF **(A)**, fALFF **(B)**, ReHo **(C)**, and DC **(D)** of six ROIs, i.e., PCC, HF, IPS, Mot, Aud, and Vis, across all subjects under eyes closed condition. TE, echo time; ALFF, amplitude of low-frequency fluctuation; fALFF, fractional ALFF; ReHo, regional homogeneity; DC, degree centrality; ROIs, regions of interest; PCC, posterior cingulate cortex; HF, left hippocampal formation; IPS, left intraparietal sulcus; Mot, left primary motor cortex; Aud, left auditory cortex; Vis, left primary visual cortex.

**FIGURE 5:**
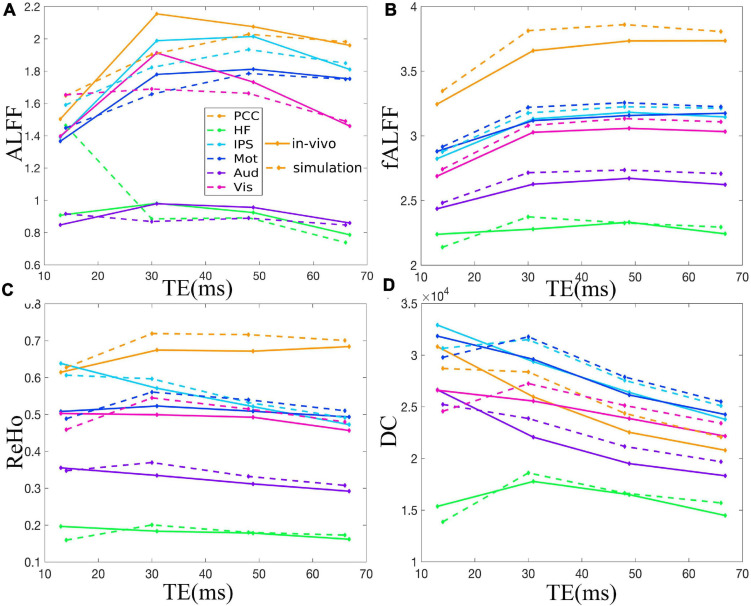
Comparison between simulation and *in vivo* value of ALFF **(A)**, fALFF **(B)**, ReHo **(C)**, and DC **(D)** of six ROIs, i.e., PCC, HF, IPS, Mot, Aud, and Vis, across all subjects under eyes closed condition. The solid and dotted lines denote the *in vivo* and simulation results, respectively. ALFF, amplitude of low-frequency fluctuation; fALFF, fractional ALFF; ReHo, regional homogeneity; DC, degree centrality; ROIs, regions of interest; PCC, posterior cingulate cortex; HF, left hippocampal formation; IPS, left intraparietal sulcus; Mot, left primary motor cortex; Aud, left auditory cortex; Vis, left primary visual cortex.

The TE dependency characteristic of the six ROIs was also analyzed at group level (Figures 4, 5 under EC condition; Supplementary Figures 1, 2 under EO condition in Supplementary Material), which indicated the similar but smaller variations across different TEs. The mean and standard deviation of the group-averaged simulated and *in vivo* ALFF, fALFF, ReHo, and DC of six ROIs across different TEs under eyes closed condition are displayed in Table 1.

**TABLE 1:** The mean and standard deviation of the group averaged simulated and *in vivo* ALFF, fALFF, ReHo, and DC of six ROIs across different TEs under eyes closed condition.

Metrics	ROIs	14 ms	30 ms	48 ms	66 ms	Metrics	ROIs	13 ms	30.9 ms	48.86 ms	66.79 ms
ALFF_simulation	PCC	1.65 ± 0.90	1.90 ± 0.81	2.03 ± 0.80	1.98 ± 0.76	ALFF_*in vivo*	PCC	1.50 ± 0.85	2.15 ± 1.0	2.08 ± 0.80	1.96 ± 0.79
	HF	1.46 ± 1.35	0.89 ± 0.62	0.89 ± 0.64	0.74 ± 0.61		HF	0.91 ± 0.65	0.98 ± 0.66	0.92 ± 0.67	0.79 ± 0.65
	IPS	1.59 ± 0.83	1.82 ± 1.06	1.93 ± 1.10	1.85 ± 1.02		IPS	1.39 ± 0.63	1.99 ± 1.20	2.01 ± 1.16	1.81 ± 0.96
	Mot	1.45 ± 0.88	1.66 ± 1.09	1.79 ± 1.20	1.75 ± 1.20		Mot	1.37 ± 0.76	1.78 ± 1.23	1.81 ± 1.23	1.75 ± 1.16
	Aud	0.92 ± 0.35	0.87 ± 0.44	0.89 ± 0.46	0.85 ± 0.43		Aud	0.85 ± 0.33	0.98 ± 0.45	0.96 ± 0.46	0.86 ± 0.41
	Vis	1.65 ± 0.79	1.69 ± 0.94	1.66 ± 0.94	1.49 ± 0.87		Vis	1.40 ± 0.61	1.91 ± 1.08	1.73 ± 0.99	1.46 ± 0.81
fALFF_simulation	PCC	3.35 ± 0.44	3.81 ± 0.42	3.86 ± 0.41	3.81 ± 0.41	fALFF_*in vivo*	PCC	3.24 ± 0.42	3.66 ± 0.43	3.73 ± 0.39	3.74 ± 0.42
	HF	2.14 ± 0.45	2.37 ± 0.57	2.33 ± 0.56	2.29 ± 0.56		HF	2.24 ± 0.50	2.28 ± 0.47	2.33 ± 0.53	2.24 ± 0.54
	IPS	2.88 ± 0.54	3.18 ± 0.62	3.23 ± 0.61	3.21 ± 0.59		IPS	2.82 ± 0.54	3.13 ± 0.61	3.18 ± 0.59	3.15 ± 0.59
	Mot	2.92 ± 0.57	3.22 ± 0.57	3.26 ± 0.57	3.22 ± 0.58		Mot	2.88 ± 0.54	3.12 ± 0.60	3.16 ± 0.58	3.17 ± 0.57
	Aud	2.48 ± 0.44	2.71 ± 0.57	2.74 ± 0.55	2.71 ± 0.51		Aud	2.44 ± 0.47	2.63 ± 0.52	2.67 ± 0.53	2.62 ± 0.48
	Vis	2.74 ± 0.58	3.08 ± 0.62	3.13 ± 0.63	3.11 ± 0.62		Vis	2.69 ± 0.52	3.03 ± 0.59	3.06 ± 0.61	3.03 ± 0.60
ReHo_simulation	PCC	0.63 ± 0.10	0.72 ± 0.10	0.72 ± 0.10	0.70 ± 0.10	ReHo_*in vivo*	PCC	0.61 ± 0.10	0.67 ± 0.11	0.67 ± 0.11	0.68 ± 0.10
	HF	0.16 ± 0.09	0.20 ± 0.12	0.18 ± 0.11	0.17 ± 0.1		HF	0.20 ± 0.12	0.18 ± 0.11	0.18 ± 0.11	0.16 ± 0.09
	IPS	0.61 ± 0.16	0.60 ± 0.13	0.53 ± 0.12	0.49 ± 0.11		IPS	0.64 ± 0.14	0.57 ± 0.13	0.52 ± 0.12	0.47 ± 0.11
	Mot	0.49 ± 0.15	0.56 ± 0.16	0.54 ± 0.15	0.51 ± 0.15		Mot	0.51 ± 0.15	0.52 ± 0.16	0.51 ± 0.16	0.49 ± 0.15
	Aud	0.35 ± 0.12	0.37 ± 0.13	0.33 ± 0.12	0.31 ± 0.12		Aud	0.36 ± 0.14	0.33 ± 0.12	0.31 ± 0.11	0.29 ± 0.11
	Vis	0.46 ± 0.18	0.55 ± 0.17	0.51 ± 0.17	0.48 ± 0.17		Vis	0.50 ± 0.17	0.50 ± 0.18	0.49 ± 0.17	0.46 ± 0.17
DC_simulation	PCC	28,714 ± 7,889	28,376 ± 9,592	24,369 ± 9,637	22,066 ± 9,060	DC_*in vivo*	PCC	30,824 ± 8,201	25,966 ± 9,921	22,533 ± 9,509	20,793 ± 8,796
	HF	13,864 ± 10,302	18,607 ± 12,118	16,639 ± 10,807	15,704 ± 9,977		HF	15,365 ± 10,905	17,779 ± 11,663	16,499 ± 10,710	14,481 ± 9,219
	IPS	30,659 ± 7,502	31,504 ± 6,836	27,559 ± 6,724	25,102 ± 6,717		IPS	32,911 ± 6,610	29,366 ± 7,476	26,395 ± 6,762	23,787 ± 6,676
	Mot	29,781 ± 8,378	31,782 ± 7,094	27,889 ± 7,223	25,496 ± 7,002		Mot	31,842 ± 8,451	29,597 ± 7,619	26,143 ± 7,608	24,255 ± 6,879
	Aud	25,231 ± 8,940	23,870 ± 9,659	21,166 ± 8,734	19,689 ± 8,017		Aud	26,642 ± 9,849	22,062 ± 9,336	19,505 ± 8,627	18,328 ± 8,042
	Vis	24,566 ± 9,751	27,251 ± 9,045	25,131 ± 8,101	23,414 ± 7,516		Vis	26,603 ± 9,876	25,557 ± 8,900	23,865 ± 8,187	22,162 ± 7,654

*TE, echo time; ALFF, amplitude of low-frequency fluctuation; fALFF, fractional ALFF; ReHo, regional homogeneity; DC, degree centrality; ROIs, regions of interest; PCC, posterior cingulate cortex; HF, left hippocampal formation; IPS, left intraparietal sulcus; Mot, left primary motor cortex; Aud, left auditory cortex; Vis, left primary visual cortex.*

### TE Dependency of *T* Maps of Local Activity Metrics

**FIGURE 6:**
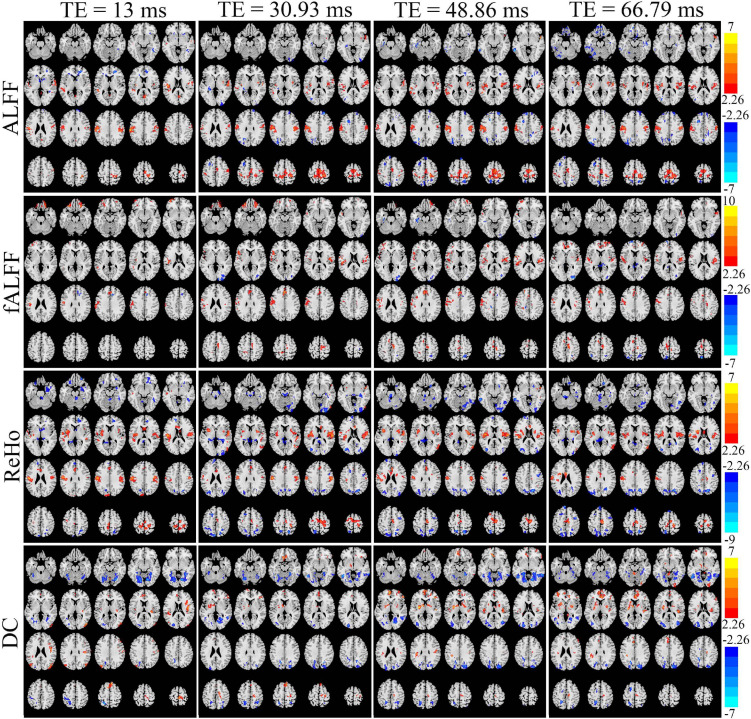
The paired *T* maps between EO and EC of ALFF, fALFF, ReHo, and DC from four different TE datasets. EO, eyes open; EC, eyes closed; ALFF, amplitude of low-frequency fluctuation; fALFF, fractional ALFF; ReHo, regional homogeneity; DC, degree centrality.

Figure 6 displays the *T* maps of local activity metrics at different TEs. For ALFF, the area size and distribution of regions with a significant difference between EO and EC altered with TE (Figure 6). The total voxel number of thresholded *T* map in the middle and lateral frontal lobe, somatosensory cortex, supplementary motor area (SMA), and lateral occipital cortex became larger with longer TE, while the total number of voxels in the temporal lobe changed little when TE became longer. For fALFF, the number of voxels with significant difference in most of the areas changed irregularly with TEs. For ReHo, the voxel number in the somatosensory cortex and middle and lateral occipital area varied little across the TEs. For DC, the significant area size and distribution of *T* maps altered greatly across the four TEs.

**FIGURE 7:**
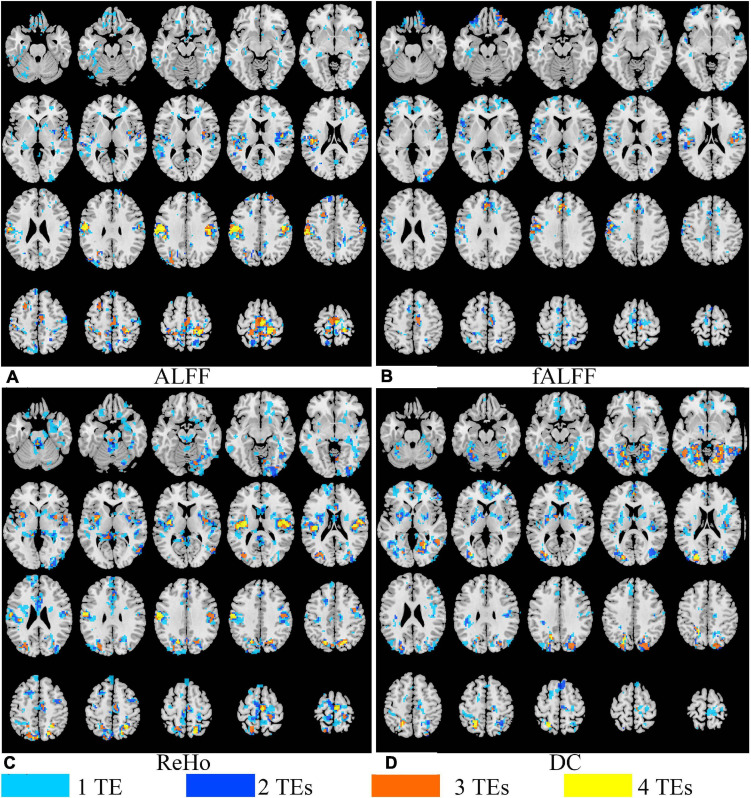
The overlap of paired *T* maps of between EO and EC from four different TE datasets for ALFF **(A)**, fALFF **(B)**, ReHo **(C)**, and DC **(D)**. “1 TE” means any one of the four echoes, “2 TEs” means any two of the four echoes, “3 TEs” means any three of the four echoes, and “4 TEs” means all the four echoes. EO, eyes open; EC, eyes closed; ALFF, amplitude of low-frequency fluctuation; fALFF, fractional ALFF; ReHo, regional homogeneity; DC, degree centrality.

Generally, all the four local activity metrics (i.e., ALFF, fALFF, ReHo, and DC) displayed different TE dependency in different ROIs (Figure 7). EO and EC difference of ALFF and ReHo across four TEs was consistently observed only in the somatosensory cortex. The DOCs of ALFF, fALFF, ReHo, and DC for voxels that appeared in the thresholded *T* maps of all the four TE datasets were only 6.87, 0.73, 5.08, and 3.80%, respectively, while the DOCs for voxels that appeared in thresholded *T* map of only one TE dataset were 56.71, 69.44, 62.26, and 62.63%, respectively (Table 2).

**TABLE 2:** The DOCs of ALFF, fALFF, ReHo, and DC for voxels that appeared in the *T* maps between EO and EC of only one TE dataset, any two TE datasets, any three TE datasets, and all the four TE datasets, respectively.

	ALFF (%)	fALFF (%)	ReHo (%)	DC (%)
1 TE	56.71	69.44	62.26	74.19
2 TEs	20.26	20.48	20.46	18.91
3 TEs	16.16	9.36	12.20	5.96
4 TEs	6.87	0.73	5.08	0.93

*DOC, Dice overlap coefficient; ALFF, amplitude of low-frequency fluctuation; fALFF, fractional ALFF; ReHo, regional homogeneity; DC, degree centrality; EO, eyes open; EC, eyes closed; TE, echo time.*

## Discussion

In this study, we explored the TE dependency of four local activity metrics (i.e., ALFF, fALFF, ReHo, and DC) with ME-fMRI. The simulated and *in vivo* results showed that all the four local activity metrics presented initial dramatic change at short TE, while displayed much flatter variation as TE became longer. Further, for the within-subject group analysis between EO and EC of the four local activity metrics, the area with significant difference varied greatly with TE. Collectively, these findings demonstrated that local metrics were greatly dependent on TE.

### TE Dependency of Local Activity Metrics

The fluctuation of fMRI signals (△*S*(*t*)) can be derived by expanding Eq. (1) with a first-order approximation for a small change in *S*_0_ and $R_{2}^{*}$ (Kundu et al., 2014):

(2) $$\begin{array}{c} △S\left(t\right)=-S_{mean}\cdot TE\cdot △R_{2}^{*}\left(t\right)+S_{mean}\cdot \frac{△S_{0}\left(t\right)}{S_{0,\text{mean}}},\\ S_{mean}=S_{0,mean}\cdot \exp\left(-TE\cdot R_{2,\text{mean}}^{*}\right), \end{array}$$

where *S*_0,mean_ and $R_{2,\text{mean}}^{*}$ are the mean values of *S*_0_(*t*) and $R_{2}^{*}\left(t\right)$ of each voxel, respectively, and △*S*_0_(*t*) and $△R_{2}^{*}\left(t\right)$ are fluctuations of *S*_0_(*t*) and $R_{2}^{*}\left(t\right)$, namely, *S*_0_(*t*)−*S*_0,mean_ and $R_{2}^{*}\left(t\right)-R_{2,\text{mean}}^{*}$, respectively. When the alteration of *S*_0_ is neglected, the relationship between BOLD contrast signal △*S*(*t*) and TE is a bell shape (Kundu et al., 2012). At TE = $T_{2}^{*}$, maximum △*S*(*t*) is achieved and the ALFF is also the maximum correspondingly.

An ALFF curve may include a descent, ascent, and then a descent trend again (Figure 2). According to Eq. (2), this is because the △*S*_0_(*t*) effect is the dominant contribution to ALFF when TE is small, whereas when TE becomes larger, the $△R_{2}^{*}\left(t\right)$ effect becomes more notable and even dominant. As a result, the ALFF curve appears to be a bell shape. In contrast, Wu and colleagues demonstrated that ALFF displayed a convex shape across all TEs (Wu et al., 2012). This difference was due to the ALFF was normalized to the signal of 0 Hz, i.e., the percentage of signal change in Wu et al. (2012) study, rather than the conventional ALFF (Zang et al., 2007; Wu et al., 2012). Moreover, the mean ALFF of an intrinsic network used in Wu’s study combined all the information of all voxels within a network, and thus, the TE dependency characteristic of each voxel was lost. Additionally, the scanning parameter with shortest TE = 10 ms (Wu et al., 2012) limited the demonstration of variation trend at very short TE, especially the initial decrease at ultrashort TE.

The fALFF reached its maxima and entered the equilibrium stage earlier than ALFF, which may be due to the intrinsic computation feature of fALFF. When TE is very short, *S*_0_(*t*) dominates the fMRI signal fluctuation (Wu and Li, 2005). By dividing the mean of whole power spectrum, fALFF suppresses the noise at short TE and normalizes the low-frequency power at long TE (Zou et al., 2008), thus avoiding very high initial value, accelerating the platform stage, and leading to more stable value at longer TEs (Wu and Li, 2005).

Both ReHo and DC reflect functional connectivity (Zang et al., 2004; Zuo et al., 2012). Specially, the ReHo is the homogeneity of a given voxel and its neighbors and represents a kind of local FC, while the DC essentially reflects the whole-brain FC of a given voxel. Our simulation and *in vivo* results were consistent with previous studies (Peltier and Noll, 2002; Wu et al., 2012). For instance, Peltier and Noll (2002) demonstrated that seed-based FC became significant in later echo times. Moreover, Wu and colleagues demonstrated that the overall FC in an intrinsic network presented a convex relationship with TE (Wu et al., 2012).

As for the group level, the TE dependency characteristic of the six ROIs (Figures 4, 5 under EC condition; Supplementary Figures 1, 2 under EO condition in Supplementary Material) indicated a similar but smaller variations across different TEs, which might be due to that the group-averaged results obscured the observation of more detailed and specific TE dependency information.

### TE Dependency of *T* Maps of Local Activity Metrics

The patterns with significant difference between EO and EC from E2 with TE = 30.93 ms were consistent with those in previous RS-fMRI studies, where single-echo EPI was used with TE approximate to 30 ms (Liu et al., 2013; Liang et al., 2014; Xu et al., 2014; Zou et al., 2015; Wei et al., 2018). For ALFF and ReHo, the areas with significant difference were consistent with previous results using TE = 27 or 30 ms (Liu et al., 2013; Zou et al., 2015), where the sensorimotor cortex, SMA, the paracentral lobe, and premotor and auditory cortex were demonstrated to be significant different areas in line with other studies with TE = 30 ms (Liang et al., 2014; Wei et al., 2018). Further, for DC, the sensorimotor cortex and occipital cortex showed a significant difference when TE was 30 ms, which agreed with previous studies (Xu et al., 2014; Wei et al., 2018).

As for the special TE dependency of the significant areas, the results could be closely related to the changes of voxel intrinsic parameters [i.e., $T_{2}^{*}\left(t\right)$ and *S*_0_(*t*)] between EO and EC. For example, as the $T_{2}^{*}$ in the whole brain covers a large range (Peltier and Noll, 2002), the TE effect on each voxel is different, which cannot be removed by the division of global mean value of each subject. For the group statistical analysis, the area difference could either increase or decrease and there was no pattern along with TE. These somewhat varying results could be due to the different domination of △*S*_0_(*t*) and $△R_{2}^{*}\left(t\right)$ between EO and EC. For instance, for ALFF, if the size of a significant area was larger at shorter TE than longer TE, the difference of △*S*_0_(*t*) may have dominated the ALFF difference; if the relationship between the size of a significant area and TE was bell shaped, then the difference of $△R_{2}^{*}\left(t\right)$ may have dominated the ALFF difference; if the size of a significant area did not affect TE, then the difference could have been attributed to both the ALFF difference of $△R_{2}^{*}\left(t\right)$ and △*S*_0_(*t*).

Local activity metrics demonstrated different sensitivity to TE. The differences in brain regions, such as SMA, somatosensory cortex, and middle frontal cortex, became larger with longer TE for ALFF and almost did not change for ReHo (Figures 6, 7). Also, the difference in regions of fALFF and DC varied greatly with different TEs, which further led to the smaller overlap of *T* maps in the four different TE datasets (Table 2). These results implied that fALFF and DC were more sensitive to TE than ALFF and ReHo.

## Limitations

In the present study, only six ROIs were selected for the simulation. Although the current results give a clear impression on the issue of how TE influences the local activity metrics, further studies will benefit from the investigation of a higher number of ROIs. Moreover, the area difference between EO and EC for fALFF and DC was relatively small, and the reliability of the TE dependency of fALFF and DC needs to be further explored in other experimental designs. Further, the current study is based on a single EO/EC dataset with a relatively small subject number, and the investigation of more and larger datasets will benefit for generalizing the findings to extensive studies. In addition, as the BOLD signal fluctuation is affected by many factors, the intersection of TE and other covariates on the local activity metrics is a question worth investigating in the future.

## Conclusion

The current study demonstrats that local activity metrics are greatly dependent on TE. Based on these new findings, we conclude that it is fundamental to carefully consider TE parameters for the optimization of data acquisition and multi-center data analysis in RS-fMRI, as a poor selection of TE can lead to false positive or false negative results and reduced reliability among studies.

## Data Availability Statement

The raw data supporting the conclusions of this article will be made available by the authors, without undue reservation.

## Ethics Statement

The studies involving human participants were reviewed and approved by the Ethics Committee at the Center for Brain Imaging Science and Technology, Zhejiang University. The patients/participants provided their written informed consent to participate in this study.

## Author Contributions

HH and LXY designed the experiment and acquired the data. NZ and LXY performed the data analysis and wrote the manuscript. YTL and XQW provided advices on the analysis and interpretation of the results. All authors contributed to the article and approved the submitted version.

## Conflict of Interest

The authors declare that the research was conducted in the absence of any commercial or financial relationships that could be construed as a potential conflict of interest.

## Footnotes

1 http://www.fil.ion.ucl.ac.uk/spm

2 http://restfmri.net/forum/DPARSF
