# Supplementary material for: Echo Time Dependency of Local Activity Metrics of Resting-State Functional MRI
Source: Front Neurosci. 2021 Mar 16;15:619412. doi: 10.3389/fnins.2021.619412 (PMC8008056; doi:10.3389/fnins.2021.619412)
Supplement: Supplementary file 1 [file Image_1.pdf]

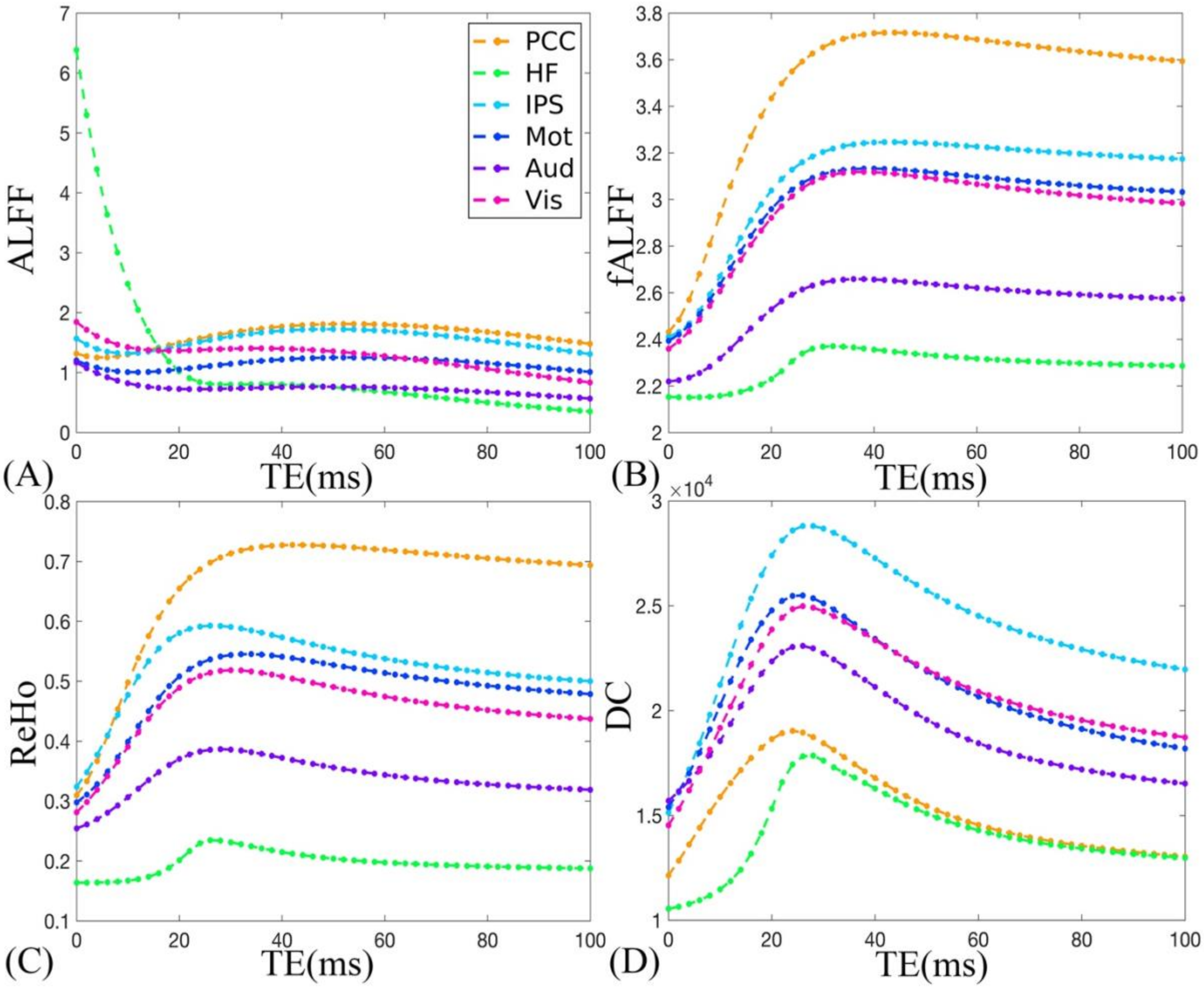

Figure 1. The simulation of the TE dependency of the group averaged ALFF (A), fALFF (B), ReHo (C), and DC (D) of six ROIs, i.e., PCC, HF, IPS, Mot, Aud, and Vis, across all subjects under eyes open condition. ALFF, the amplitude of low frequency fluctuation; fALFF, factional ALFF; ReHo, regional homogeneity; DC, degree centrality; PCC, the posterior cingulate cortex; HF, the left hippocampal formation; IPS, the left intraparietal sulcus; Mot, the primary motor cortex; Aud, the left auditory cortex; Vis, the left primary visual cortex.

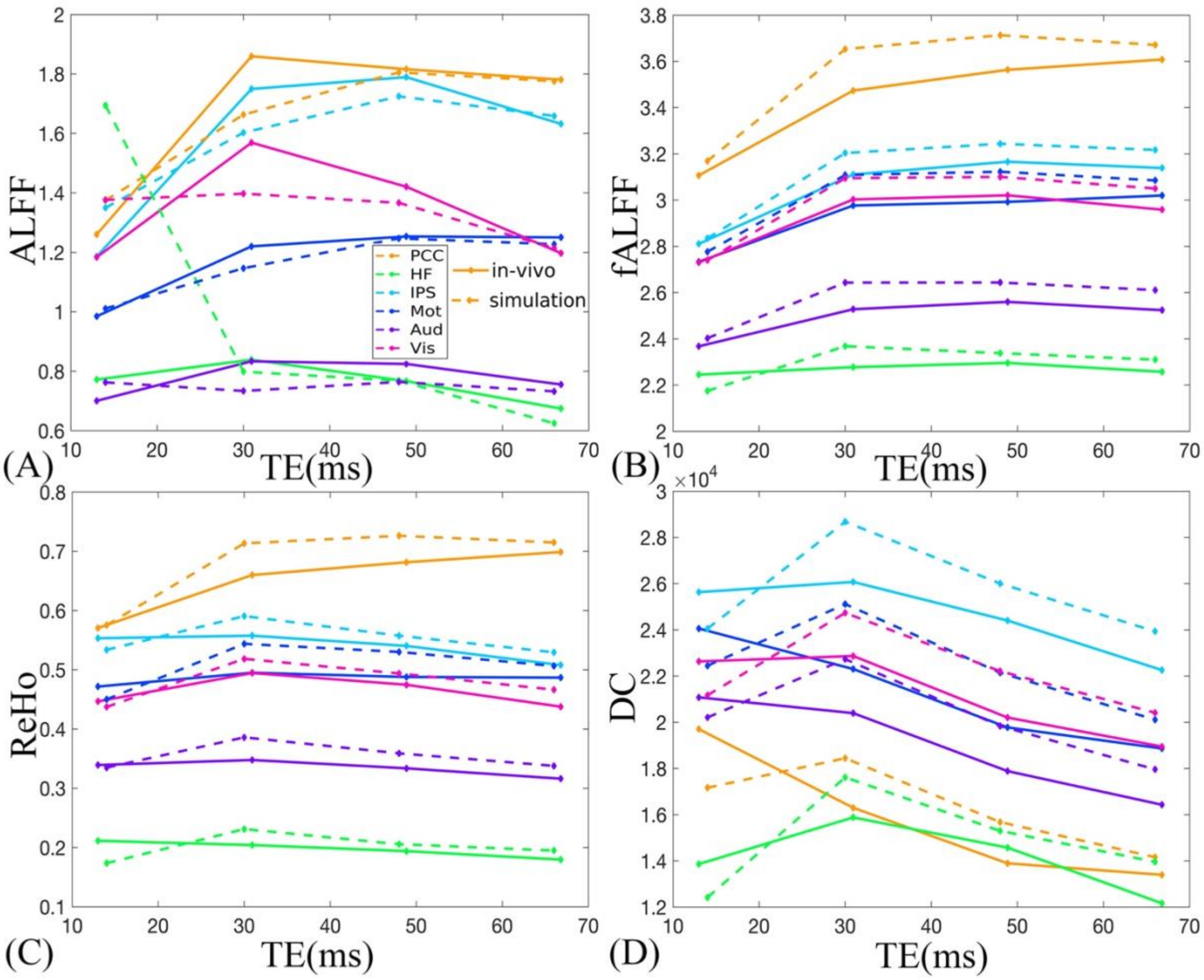

Figure 2. Comparison between simulation and in-vivo value of ALFF (A), fALFF (B), ReHo (C), and DC (D) of six ROIs, i.e., PCC, HF, IPS, Mot, Aud, and Vis, across all subjects under eyes open condition. The solid and dotted lines denoted the in-vivo and simulation results, respectively. ALFF, the amplitude of low frequency fluctuation; fALFF, factional ALFF; ReHo, regional homogeneity; DC, degree centrality; PCC, the posterior cingulate cortex; HF, the left hippocampal formation; IPS, the left intraparietal sulcus; Mot, the primary motor cortex; Aud, the left auditory cortex; Vis, the left primary visual cortex.
